# Assessment of TNF‐α (‐857 C/T) gene polymorphism in oral lichen planus disease: A case–control study

**DOI:** 10.1002/hsr2.2014

**Published:** 2024-04-03

**Authors:** Mohammad Hesam Marabi, Kheirollah Yari, Hamid Reza Mozaffari, Masoud Hatami

**Affiliations:** ^1^ Molecular Genetics Laboratory, Medical Biology Research Center, Health Technology Institute Kermanshah University of Medical Sciences Kermanshah Iran; ^2^ Department of Oral and Maxillofacial Medicine, School of Dentistry Kermanshah University of Medical Sciences Kermanshah Iran

**Keywords:** genetic polymorphism, OLP, PCR‐CTPP, SNP, TNF‐α

## Abstract

**Background and aims:**

Oral lichen planus (OLP) is an inflammatory mucocutaneous disorder with an immune‐mediated pathogenesis. The tumor necrosis factor‐α (TNF‐α) level in the serum of OLP patients is significantly higher than in the control group. TNF‐α‐857 C/T polymorphism can be related to the increased TNF‐α level in blood circulation. This study investigated the relationship between TNF‐α (‐857 C/T) polymorphism and OLP patients in an Iranian population.

**Methods:**

Saliva samples were taken from 200 people, including 100 patients with OLP and 100 healthy people who did not have significant differences in age and sex. Then, DNA was extracted from them and the TNF‐α (‐857 C/T) genotype was identified using the polymerase chain reaction with confronting two‐pair primers method. Statistical Package for the Social Sciences version 16 software analyzed the results.

**Results:**

The frequency of C/C, C/T, and T/T genotypes of the TNF‐α‐857 C/T polymorphism in the patient group were 78%, 18%, and 4%, respectively, and in the control group were 72%, 23%, and 5%, respectively. The differences between the two groups regarding allele (*χ* ^2^  =  0.97, *p*  = 0.32) and genotype (*χ* ^2^  =  0.96, *p*  = 0.62) frequency among the studied population were insignificant.

**Conclusion:**

This study showed that the difference in the frequency of single nucleotide polymorphism TNF‐α‐857 C/T polymorphism in the patient and control group had no significant relationship with the increased OLP incidence. Also, no significant association was observed between allele and genotype frequency of TNF‐α (‐857 C/T) with OLP subtypes.

## 1 INTRODUCTION

Lichen planus is a mucocutaneous disorder characterized by abnormal epithelial keratinization cycles and T‐cell‐mediated immune responses.^1^ Oral lichen planus (OLP) appears as white lesions like striations, papules, and plaques, and red lesions like erythema, abrasions, or blisters, mainly affecting the tongue, buccal mucosa, and gingiva. Although it sometimes involves other places. Lesions are usually bilateral and often present as a combination of clinical symptoms.^2^, ^3^ OLP occurs more than the cutaneous form. It is also more lasting and resistant to treatment.^4^ OLP is usually seen in adults’ fifth to sixth decade of life; it is rarely seen in children.^5^ OLP is twice as common in women as in men.^6^ Clinically, it may be categorized into different types, such as papular, atrophic, plaque‐like, reticular, erosive, and bullous.^7^

The most common forms of OLP are reticular and erosive.^8^ As OLP has the risk of developing into oral squamous cell carcinoma (OSCC), it is therefore defined as a potentially malignant disease.^9^, ^10^ Atrophic and erosive forms of OLP and plaque lesions on the back of the tongue are more potent to become malignant.^5^ For detecting OSCC, saliva tumor necrosis factor‐α (TNF‐α) is a better sample. Salivary TNF‐α levels increase in the histological differentiation grade in OSCC.^11^ Studies show that factors such as stress, drugs, genetics, dental materials, liver diseases, hepatitis C virus infection, tobacco, and alcohol can be effective in causing OLP.^5^, ^12^, ^13^, ^14^ OLP is an autoimmune T‐cell‐mediated disease, but its cause is often unknown. There is a theory about immune responses mediated by T cells that play a role in it.^15^ A vital and early condition in OLP is the increased level of TH1 cytokines, which is genetically induced, and the resulting lesions in the mouth and skin appear to be due to genetic polymorphism of the cytokines.^16^ TNF‐α, as a pro‐inflammatory cytokine, plays a critical role in the pathogenesis of numerous inflammatory or autoimmune disorders. TNF‐α is extensively reported in OLP pathogenesis compared with other inflammation‐related cytokines.^17^, ^18^ The level of TNF‐α in the serum of OLP patients was significantly higher than the control group.^9^, ^19^ TNF‐α expression level and genetic polymorphisms are involved in many diseases.^2^ Several single nucleotide polymorphisms (SNPs) that play a role in transcriptional regulation have been reported in the promoter region of the *TNF‐α* gene.^20^ One of these is SNP at position ‐857. The association between this C/T SNP and autoimmune disorders like Behçet's disease has been shown in several studies.^21^ The effect of this polymorphism is through the regulation of TNF‐α gene transcription and, as a result, its expression level. As a result, this polymorphic change can be related to the high level of TNF‐α in the blood circulation.^2^, ^22^

Reported studies confirmed that TNF‐α is a highly polymorphic gene; the review of the previous studies on other polymorphisms of TNF‐a sites and other cytokines generally suggests a relationship with the occurrence of OLP disease. However, there is no study on the association of TNF‐α‐857 polymorphism in patients with OLP. Therefore, our study aims to address this knowledge gap and explore the potential link between TNF‐α‐857 polymorphism and OLP in the Iranian population (Kermanshah province of Western Iran).

## 2 METHODS

### 2.1 Study group and sampling

A total of 200 samples, including 100 patients with OLP (29 men and 71 women; 65 erosive and 35 non‐erosive) and 100 healthy individuals (28 men and 72 women) without any history of the disease, were included in our study. All studied individuals were from the Kermanshah province of Western Iran. Our groups were matched by gender and age. The average age of our study population in both groups was 48 years and most patients were 45–65 years old. The OLP diagnosis was based on the World Health Organization's definition of OLP and confirmed by pathology reports and clinical manifestations detected by an oral medicine specialist. We excluded pregnant women, OLP patients with accompanying malignancies, autoimmune/inflammatory or systemic disorders, and patients who received any relevant treatment over the past 3 months. Additionally, patients with amalgam lichenoid reaction lesions were excluded. A family history of OLP was not considered in the selection of subjects. The purpose of the research was explained to the subjects and they filled out consent forms. To maximize DNA yield, individuals were told not to eat, drink, smoke, use mouthwash, or brush their teeth for at least 2 h before taking a saliva sample. Without intervention, 3 mL of unstimulated saliva samples were taken from each individual with the spitting method^23^ in falcon tubes (15 mL). Then, collected samples were immediately transferred to the laboratory on ice and stored at −80°C until use.

### 2.2 Genetic analysis

DNA extraction from saliva samples was performed using the standard kit for extracting DNA from the saliva of Azma Elixir Pajoh Company according to its protocol. Extracted DNA was evaluated on 1% agarose gel electrophoresis. Also, the quantity and purity of the extracted DNA were analyzed using a NanoDrop (Thermo‐2000/2000c).^24^ Polymerase chain reaction (PCR) with confronting two‐pair primer technique was used to determine the genotype and all frequencies of the TNF‐α‐857 C/T. PCR amplified *TNF‐α* gene with a set of primers that are shown in Table 1.^25^ PCR parameters were as follows: one cycle at 95°C for 5 min, 30 cycles including 95°C for 1 min, 66°C for 1 min, 72°C for 1 min, and a final at 72°C for 5 min.

**Table 1:** Used primers for analysis of TNF‐α‐857 C/T with the PCR‐CTPP method.

F1: 5′‐GGG AGC TCC TGG GAG ATA TGG‐3′
R1: 5′‐CCT CTA CAT GGC CCT GTC TTG‐3′
F2: 5′‐AGT ATG GGG ACC CCC CCT TAA T‐3′
R2: 5′‐TCT GAC CCG GA G ACT CAT AAT GC‐3′

Abbreviations: PCR‐CTPP, polymerase chain reaction with confronting two‐pair primer; TNF‐α, tumor necrosis factor‐α.

**Figure 1:**
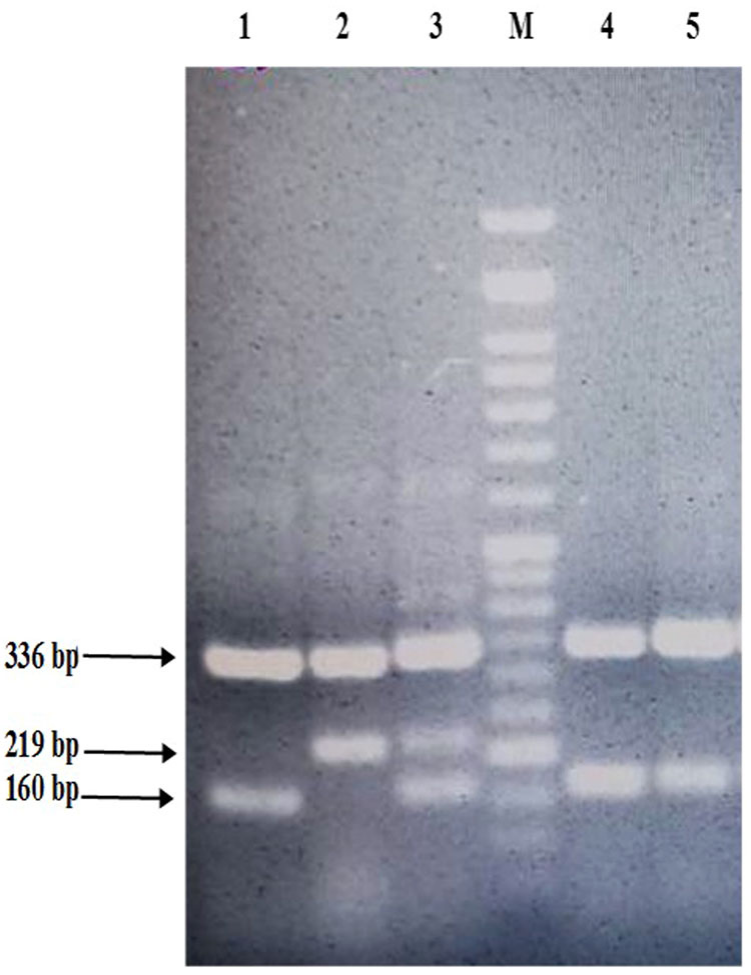
Electrophoresis pattern of polymerase chain reaction with confronting two‐pair primer (PCR‐CTPP) products for position ‐857 of *TNF‐α* gene. Lane 1: sample with T/T genotype; Lane 2: sample with C/C genotype; Lane 3: sample with T/C genotype; Lane M: DNA size marker (50 bp).

Finally, the PCR products were electrophoresed on the 2% agarose gel. In the obtained electrophoresis pattern, the CC genotype is 219 and 336 bp, the mutant genotype (TT) is 336 and 160 bp fragment, and TC as heterogeneous genotype is 336, 219, and 160 bp (Figure 1).

### 2.3 Statistical analysis

Using Statistical Package for the Social Sciences software (version 16), the *χ* ^2^ test investigated the significance of the difference in the frequency distribution of alleles and genotypes between the control and patient groups. The 95% confidence interval and the odds ratio of the logistic regression model were used. Disparities in demographic, anthropometric, and clinical characteristics between the case and control groups were assessed. Data related to quantitative characteristics are expressed as mean ± SD. The results were considered statistically significant if *p* < 0.05.

### 2.4 Ethical considerations

The Ethics Committee at Kermanshah University of Medical Sciences, Iran, approves the study design and protocols (IR.KUMS.REC.1401.386). All procedures performed were in accordance with the ethical standards and relevant guidelines and regulations in the Declaration of Helsinki. All participants were required to sign a written consent form and were provided with a participant information statement that thoroughly explained the consent process.

## 3 RESULTS

Case and control groups were matched by gender and age. The most significant number of patients was in the age range of 45–65 years (average age of 48 years). Among the OLP patients, there were 71 females and 29 males. Among 100 OLP patients, 655 were erosive and 355 of them were non‐erosive. The genotype distributions of TNF‐α‐857 in OLP patients and control groups were in Hardy–Weinberg equilibrium. The distribution of TNF‐α‐857 alleles and genotypes in OLP and control groups are depicted in Table 2. The frequencies of C/C, C/T, and T/T genotypes of the TNF‐α‐857 in the patient group were 78%, 18%, and 4%, respectively, and in the control group, were 72%, 23%, and 5%, respectively (Table 2). The results indicated no significant difference in the genotype frequencies of the codominant, dominant, overdominant, and recessive genetic models between the OLP patient and controls. As demonstrated in Table 3, no positive association was found between the frequency distribution of TNF‐α‐857 alleles and genotypes and OLP severity.

**Table 2:** Comparison of the frequency distribution of TNF‐α‐857 genotypes and alleles in OLP patients and control group.

TNF‐α‐857	Genotypes	Patient *n* = 100	Control *n* = 100	*χ* ^2^	*p*
Codominant	C/C	78	72	0.96	0.62
	C/T	18	23		
	T/T	4	5		
Dominant	C/C	78	72	0.96	0.33
	C/T + T/T	22	28		
Recessive	T/T	4	5	0.11	0.73
	C/C + C/T	96	95		
Overdominant	C/T	18	23	0.76	0.38
	T/T + C/C	82	77		
Alleles				
	C	174	167		
	T	26	33	0.97	0.32

Abbreviations: OLP, oral lichen planus; TNF‐α, tumor necrosis factor‐α.

**Table 3:** Frequencies of TNF‐α‐857 polymorphism in erosive and non‐erosive subtypes of OLP.

TNF‐α (‐857 C/T)	Erosivegroup *n* = 65	Nonerosive group *n* = 35	*χ*2	*p*
Codominant			2.34	0.31	
Wild (CC)	49	29			
Hetrozygote (CT)	12	6			
Mutant (TT)	4	0			
Dominant					
CC	49	29			
CT + TT	16	6	0.74	0.39	
Overdominant					
CT	12	6			
CC + TT	53	29	0.027	0.87	
Recessive					
CC + CT	61	35			
TT	4	0	2.24	0.13	
Allele					
C	110	64			
T	20	6	1.87	0.17	

Abbreviations: OLP, oral lichen planus; TNF‐α, tumor necrosis factor‐α.

## 4 DISCUSSION

In this study, we investigated TNF‐α gene polymorphism in the saliva sample of OLP disorder. In genomic applications, there is a need for suitable samples and efficient methods for obtaining a high yield of DNA. Saliva is an attractive source of genomic DNA because it has many advantages compared to blood, wound tissue, and different body fluid samples, such as easy access, noninvasiveness, safety, lower infection rate, and cost‐efficiency.^26^ Zhang et al.^27^ evaluated the nuclear factor‐κB‐dependent pro‐inflammatory cytokines, including TNF‐α, interleukin‐6 (IL‐6), and IL‐8 in serum and the saliva samples of OLP patients and healthy individuals; they suggested that saliva‐based test can be used as a cost‐effective adjunctive tool for measuring pro‐inflammatory cytokines in OLP patients.

In our study, the age range of 45–65 years was the most significant number of patients, which confirms the previous findings that the incidence of OLP was higher in the fourth decade of life and women.^6^, ^28^ Consistent with our results, Al‐Mohaya et al.^29^ have found that Saudi OLP patients were more frequent in the third to fourth decade of life with a median age of 42 years. In our study, 71% of OLP patients were women. This data is compatible with previous studies that show a higher prevalence of OLP among women in different populations.^29^, ^30^

It has been demonstrated that gene polymorphism in regulatory sequences of the *TNF‐α* gene can change its expression.^17^ Abnormal expression of TNF‐α in lesions, saliva, or serum of OLP patients indicates that the elevated expression of TNF‐α may be associated with OLP immunopathogenesis. In our study, we investigated the TNF‐α (‐857 C/T), because it is one of the most frequent polymorphisms in the *TNF‐α* gene that can affect the expression level of TNF‐α. The current study showed that the difference in the allele frequency of SNP TNF‐α‐857 C/T polymorphism in the control and patient group had no significant relationship with the increase in the incidence of OLP disease in the population of western Iran. Al‐Mohaya et al.^29^ concluded that TNF‐α (‐308 G/A) polymorphism is associated with OLP susceptibility among Saudi patients; also, their results support the role of genetic factors in OLP disease.

Several studies have shown the association between TNF‐α (‐857 C/T) and autoimmune or inflammatory disorders.^31^ However, the relationship between this polymorphism and OLP disease has not yet been investigated.^20^, ^21^ Limited studies with contradictory results have been done on other TNF‐α gene polymorphisms with inflammatory diseases. The association between TNF‐α‐857 polymorphism and ulcerative colitis and Crohn's disease has been investigated, and a significant relationship with Crohn's disease was observed.^32^ A systematic review and meta‐analysis by Zhou and Vieira^33^ showed that TNF‐α ‐308 G/A polymorphism in TNF‐α is a potential genetic marker for OLP. Kimkong et al.^34^ found no significant correlation between TNF‐α polymorphisms (‐863 and ‐238) and OLP development in a Thai population; however, a higher proportion of TNF‐α‐308 AA genotype) among OLP patients compared to healthy controls was reported. In addition, we studied the effect of TNF‐α (‐857 C/T) gene polymorphism on OLP severity was studied. Our results indicate no significant difference between the TNF‐α (‐857 C/T) allele and genotype frequency with OLP subtypes. Our result is consistent with a previous report, which found no significant association between TNF‐α gene polymorphism (‐863 and ‐238) and the development of OLP in the Thai population.^34^ In another study, Carrozzo et al.^35^ investigated 13 cytokine genes with 22 SNPs on sensitivity to Caucasian OLP. They suggested that the increased frequency of the –308 A TNF‐α allele and genetic polymorphism of the first intron of the *interferon‐γ* gene may contribute to the development of OLP lesions.

## 5 CONCLUSION

Our study confirms that OLP in the Kermanshah province of Western Iran is present mainly in the fourth decade of life, between 45 and 65 years old. Our results indicated no positive association between allele and genotype frequencies of SNP TNF‐α‐857 C/T polymorphism and incidence/severity of OLP disease in the population of western Iran (Kermanshah province). More studies with larger sample sizes, evaluations of TNF‐α gene expression, and haplotyping with different SNPs are needed to understand better the role of the TNF‐α‐857 C/T in the pathogenesis and severity of OLP disease.

## AUTHOR CONTRIBUTIONS

**Mohammad Hesam Marabi**: Methodology, writing—original draft, data curation, project administration. **Kheirollah Yari**: Conceptualization, methodology, writing—review and editing, formal analysis, validation, supervision, project administration. **Hamid Reza Mozaffari**: Conceptualization, writing—review and editing. **Masoud Hatami**: Writing—review and editing, investigation, methodology.

## CONFLICT OF INTEREST STATEMENT

The authors declare no conflict of interest.

## ETHICS STATEMENT

The Ethics Committee at Kermanshah University of Medical Sciences, Iran, approves the current study (IR.KUMS.REC.1401.386). All participants were required to sign a written consent form and were provided with a participant information statement that thoroughly explained the consent process.

## TRANSPARENCY STATEMENT

The lead author Kheirollah Yari affirms that this manuscript is an honest, accurate, and transparent account of the study being reported; that no important aspects of the study have been omitted; and that any discrepancies from the study as planned (and, if relevant, registered) have been explained.

## Data Availability

The authors confirm that the data supporting the findings of this study are available within the article (and/or) its supplementary materials.

## References

[1] Roopashree MR , Gondhalekar RV , Shashikanth MC , George J , Thippeswamy SH , Shukla A . Pathogenesis of oral lichen planus–a review. J Oral Pathol. 2010;39(10):729‐734. 10.1111/j.1600-0714.2010.00946.x 20923445

[2] Marabi MH , Mozaffari HR , Ghasemi H , Hatami M , Yari K . Evaluation of the association between TNF‐α‐1031 T/C polymorphism with oral lichen planus disease. BMC Oral Health. 2024;24(1):189. 38317095 10.1186/s12903-024-03939-x PMC10845614

[3] Ghasemi H , Mozaffari HR , Kohsari M , Hatami M , Yari K , Marabi MH . Association of interleukin‐8 polymorphism (+ 781 C/T) with the risk of oral lichen planus disease. BMC Oral Health. 2023;23(1):404. 37340381 10.1186/s12903-023-03088-7 PMC10280860

[4] Mollaoglu N . Oral lichen planus: a review. Br J Oral Maxillofac Surg. 2000;38(4):370‐377. 10922170 10.1054/bjom.2000.0335

[5] Boorghani M , Gholizadeh N , Taghavi Zenouz A , Vatankhah M , Mehdipour M . Oral lichen planus: clinical features, etiology, treatment and management; a review of literature. J Dent Res Dent Clin Dent Prospects. 2010;4(1):3‐9. 22991586 10.5681/joddd.2010.002 PMC3429956

[6] Scully C , Carrozzo M . Oral mucosal disease: lichen planus. Br J Oral Maxillofac Surg. 2008;46(1):15‐21. 17822813 10.1016/j.bjoms.2007.07.199

[7] Lodi G , Scully C , Carrozzo M , Griffiths M , Sugerman PB , Thongprasom K . Current controversies in oral lichen planus: report of an international consensus meeting. Part 1. Viral infections and etiopathogenesis. Oral Surg Oral Med Oral Pathol Oral Radiol Endodontol. 2005;100(1):40‐51. 10.1016/j.tripleo.2004.06.077 15953916

[8] Sharma S , Saimbi C , Koirala B . Erosive oral lichen planus and its management: a case series. JNMA J Nepal Med Assoc. 2008;47(170):86‐90. 18709038

[9] Rhodus NL , Cheng B , Myers S , Bowles W , Ho V , Ondrey F . A comparison of the pro‐inflammatory, NF‐κB‐dependent cytokines: TNF‐alpha, IL‐1‐alpha, IL‐6, and IL‐8 in different oral fluids from oral lichen planus patients. Clin Immunol. 2005;114(3):278‐283. 15721838 10.1016/j.clim.2004.12.003

[10] Sun A , Wang JT , Chia JS , Chiang CP . Serum interleukin‐8 level is a more sensitive marker than serum interleukin‐6 level in monitoring the disease activity of oral lichen planus. Br J Dermatol. 2005;152(6):1187‐1192. 15948980 10.1111/j.1365-2133.2005.06497.x

[11] G D , Nandan SRK , Kulkarni PG . Salivary tumour necrosis factor‐α as a biomarker in oral leukoplakia and oral squamous cell carcinoma. Asian Pacific J Cancer Prev. 2019;20(7):2087‐2093. 10.31557/APJCP.2019.20.7.2087 PMC6745219 31350970

[12] Mozaffari HR , Rahmani M , Rezaei F , Sadeghi M , Sharifi R , Ejtehadi A . Evaluation of oral lichen planus frequency in patients referred to pathology centers of Kermanshah city, during 2008 to 2011. Scholars J Appl Med Sci. 2016;4(6E):2200‐2202.

[13] Ismail SB , Kumar SKS , Zain RB . Oral lichen planus and lichenoid reactions: etiopathogenesis, diagnosis, management and malignant transformation. J Oral Sci. 2007;49(2):89‐106. 17634721 10.2334/josnusd.49.89

[14] Ghasemi H , Mozaffari HR , Kohsari M , Hatami M , Yari K , Marabi MH . Association of interleukin‐8 polymorphism (+ 781 C/T) with the risk of oral lichen planus disease. BMC Oral Health. 2023;23(1):404. 37340381 10.1186/s12903-023-03088-7 PMC10280860

[15] DeAngelis LM , Cirillo N , McCullough MJ . The immunopathogenesis of oral lichen planus‐is there a role for mucosal associated invariant T cells? J Oral Pathol Med. 2019;48(7):552‐559. 31172572 10.1111/jop.12898

[16] Liu W , Li M , Zhang X , Zhou Z , Shen Z , Shen X . Association of polymorphisms in Th1/Th2‐related cytokines (IFN‐γ, TGFβ1, IL‐1β, IL‐2, IL‐4, IL‐18) with oral lichen planus: A pooled analysis of case‐control studies. J Dental Sci. 2023;18(2):560‐566. 10.1016/j.jds.2022.08.032 PMC10068379 37021277

[17] Lu R , Zhang J , Sun W , Du G , Zhou G . Inflammation‐related cytokines in oral lichen planus: an overview. J Oral Pathol Med. 2015;44(1):1‐14. 24329772 10.1111/jop.12142

[18] Darvishi E , Aziziaram Z , Yari K , et al. Lack of association between the TNF‐α‐1031genotypes and generalized aggressive periodontitis disease. Cell Mol Biol. 2016;62(11):63‐66. 27755954

[19] Kaur J , Jacobs R . Proinflammatory cytokine levels in oral lichen planus, oral leukoplakia, and oral submucous fibrosis. J Korean Assoc Oral Maxillofac Surg. 2015;41(4):171. 26339574 10.5125/jkaoms.2015.41.4.171 PMC4558184

[20] Waldron‐Lynch F , Adams C , Amos C , et al. Tumour necrosis factor 5′ promoter single nucleotide polymorphisms influence susceptibility to rheumatoid arthritis (RA) in immunogenetically defined multiplex RA families. Genes Immun. 2001;2(2):82‐87. 11393661 10.1038/sj.gene.6363738

[21] Radouane A , Oudghiri M , Chakib A , Bennani S , Touitou I , Barat‐Houari M . SNPs in the TNF‐α gene promoter associated with Behcet's disease in Moroccan patients. Rheumatology. 2012;51(9):1595‐1599. 22711844 10.1093/rheumatology/kes141

[22] Agha‐Hosseini F , Moosavi M‐S , Sheykhbahaei N . Association of oral lichen planus and its treatment on tumor necrosis factor‐alpha: a review literature and meta‐analysis. Middle East J Rehabil Health Stud. 2021;8(3):e109577.

[23] Navazesh M . Methods for collecting saliva. Ann NY Acad Sci. 1993;694:72‐77. 8215087 10.1111/j.1749-6632.1993.tb18343.x

[24] Jalilvand A , Yari K , Aznab M , Rahimi Z , Salahshouri Far I , Mohammadi P . A case‐control study on the SNP309T → G and 40‐bp Del1518 of the MDM2 gene and a systematic review for MDM2 polymorphisms in the patients with breast cancer. J Clin Lab Anal. 2020;34(12):e23529. 32951271 10.1002/jcla.23529 PMC7755803

[25] Hamajima N , Shibata A , Katsuda N , et al. Subjects with TNF‐A‐857TT and‐1031TT genotypes showed the highest *Helicobacter pylori* seropositive rate compared with those with other genotypes. Gastric Cancer. 2003;6:230‐236. 14716517 10.1007/s10120-003-0258-z

[26] Zayats T , Young TL , Mackey DA , Malecaze F , Calvas P , Guggenheim JA . Quality of DNA extracted from mouthwashes. PLoS One. 2009;4(7):e6165. 19582144 10.1371/journal.pone.0006165 PMC2701599

[27] Zhang Y , Lin M , Zhang S , et al. NF‐κB‐dependent cytokines in saliva and serum from patients with oral lichen planus: a study in an ethnic Chinese population. Cytokine. 2008;41(2):144‐149. 18222093 10.1016/j.cyto.2007.11.004

[28] Sugerman PB , Savage NW , Walsh LJ , et al. The pathogenesis of oral lichen planus. Crit Rev Oral Biol Med. 2002;13(4):350‐365. 12191961 10.1177/154411130201300405

[29] Al‐Mohaya MA , Al‐Harthi F , Arfin M , Al‐Asmari A . TNF‐α, TNF‐β and IL‐10 gene polymorphism and association with oral lichen planus risk in Saudi patients. J Appl Oral Sci. 2015;23(3):295‐301. 26221924 10.1590/1678-775720150075 PMC4510664

[30] Bermejo‐Fenoll A , Sánchez‐Siles M , López‐Jornet P , Camacho‐Alonso F , Salazar‐Sánchez N . A retrospective clinicopathological study of 550 patients with oral lichen planus in south‐eastern Spain. J Oral Pathol Med. 2010;39(6):491‐496. 20456611 10.1111/j.1600-0714.2010.00894.x

[31] Balkrishna A , Solleti SK , Singh H , Singh R , Sharma N , Varshney A . Biotite‐calx based traditional Indian medicine Sahastraputi‐Abhrak‐Bhasma prophylactically mitigates allergic airway inflammation in a mouse model of asthma by amending cytokine responses. J Inflamm Res. 2021;14:4743‐4760. 34557016 10.2147/JIR.S313955 PMC8455516

[32] Kafshboran HR , et al Association of TNF‐α‐857 polymorphism with inflammatory bowel disease in a group of Iranian Azeri individuals. Middle East J Dig Dis. 2014;6(1):28. 24829702 PMC4005480

[33] Zhou Y , Vieira AR . Association between TNFα‐308 G/A polymorphism and oral lichen planus (OLP): a meta‐analysis. J Appl Oral Sci. 2018;26:e20170184. 29641751 10.1590/1678-7757-2017-0184 PMC5912397

[34] Kimkong I , Hirankarn N , Nakkuntod J , Kitkumthorn N . Tumour necrosis factor‐alpha gene polymorphisms and susceptibility to oral lichen planus. Oral Dis. 2011;17(2):206‐209. 20796230 10.1111/j.1601-0825.2010.01722.x

[35] Carrozzo M , Uboldi de capei M , Dametto E , et al. Tumor necrosis factor‐α and interferon‐γ polymorphisms contribute to susceptibility to oral lichen planus. J Invest Dermatol. 2004;122(1):87‐94. 14962095 10.1046/j.0022-202X.2003.22108.x

